# Editorial: Immunogenicity and toxicity of AAV gene therapy

**DOI:** 10.3389/fimmu.2023.1227231

**Published:** 2023-06-22

**Authors:** Allison Bradbury, David Markusic, Manish Muhuri, Li Ou

**Affiliations:** ^1^ Center for Gene Therapy, Abigail Wexner Research Institute Nationwide Children’s Hospital and Department of Pediatrics, The Ohio State University, Columbus, OH, United States; ^2^ Department of Pediatrics, Indiana University, Indianapolis, IN, United States; ^3^ Horae Gene Therapy Center, Department of Microbiology and Physiological Systems, University of Massachusetts Chan Medical School, Worcester, MA, United States; ^4^ Department of Research & Discovery, Genemagic Biosciences, Philadelphia, PA, United States; ^5^ Department of Pediatrics, University of Minnesota, Minneapolis, MN, United States

**Keywords:** adeno-associated virus, gene therapy, immune responses, toxicity, immune evasion, durability, neutralizing antibodies

Recombinant adeno-associated virus (AAV) is a promising delivery vehicle for *in vivo* gene therapy and has been widely tested in preclinical and clinical studies. However, despite the vast promise they hold for gene therapy, debilitating immune responses have been reported against the AAV capsid itself and/or to the transgene product. As a result of high vector doses, serious adverse effects like complement activation and liver toxicity have been observed, even leading to fatality of subjects in certain clinical trials. Although many of these adverse events can be managed with immunosuppressants, this is not true for all trial participants. To gain a better understanding of factors that may influence AAV immunity and durability, Shen et al. conducted a meta-analysis of 255 AAV clinical trials over the past 25 years. The usage of immunosuppressants and antibody screening assays highly depended on the indications, with relatively lower frequency among trials for treating retinal and central nervous system (CNS) diseases. The rates of treatment emergent serious adverse events (TESAEs) also varied by indication, routes of administration, serotype, and dosage, with CNS diseases having the highest risk. Other interesting findings of their analysis include a broad range of AAV neutralizing antibody titer cutoffs between trials and that the vector production platform had no impact on gene therapy durability or frequency of adverse events.

Recently, complement-mediated AEs have emerged as contributing to immune-mediated toxicities, specifically in high-dose systemic delivery of AAV vectors. Because animal models have not shown a direct role of complement in AAV mediated toxicities, detailed mechanistic studies are lacking. To address this knowledge gap, Smith et al. conducted studies in human PBMCs obtained from multiple donors and found that high AAV neutralizing antibody titers >1:100 enhanced the uptake of AAV viral particles (empty or full) in several immune cells including neutrophils, monocyte-related dendritic cells, and monocytes leading to increased production of pro-inflammatory cytokines/chemokines and complement activation. Uptake and immune cell activation by AAV vectors were significantly reduced with a C3 inhibitor, APL-9.

While immune responses to AAV capsids have been extensively characterized, detrimental responses to the transgene product have been less frequently reported. Hordeaux et al. developed an AAV-mediated gene replacement therapy for Pompe disease that consists of an AAVhu68 capsid, insulin-like growth factor 2 variant (vIGF2) peptide enhancer, and a human acid-alpha glucosidase (hGAA) transgene. The gene therapy was systemically delivered to rhesus macaques in a dose escalation study: 1x10^13^ genome copies (GC)/kg, 5x10^13^GC/kg, or 1x10^14^ GC/kg. At the study endpoint of day 60, 5 animals (1 low dose and 4 high doses) had elevated troponin I levels, a marker of myocardial damage, which correlated with histopathological findings in the heart post-mortem. Cardiac infiltrates were found to be T-cell rich and 3 of the macaques with cardiac toxicity had higher ELISPOT response to hGAA peptide pools. Together, these findings suggest cardiac toxicity primarily caused by a cytotoxic T-cell-mediated response to the hGAA transgene in cardiomyocytes. Although variability was noted in this study, an association between an MHC class I haplotype and toxicity was found in 3 animals. In addition to the clear safety concern, transgene-mediated immunity is likely to dramatically impact therapeutic efficacy and durability. Differences in MHC haplotypes and reactivity to human transgenes in preclinical animal models should be further explored and considered.

Additionally, in a perspective article, Ertl has addressed the different types of immune responses that are elicited by AAVs and how they translate into a lack of efficacy. This article provides mechanistic insights into how innate responses, complement responses, and B- and T-cell responses are activated by AAV administration along with highlighting some of the adverse immunotoxicity-related events caused by high-dose AAV gene transfer.

Extensive efforts are underway to circumvent the activation of these immune response pathways by research groups across the field. Li et al. have provided a holistic overview of these endeavors in a review article. The authors have generated a database or toolkit that will serve as a resource for methods to overcome AAV immunity that are categorized mainly based on methodology, instead of on targets. Both the preclinical data as well as the clinical data, if available, have been discussed for each strategy. Some of the strategies covered here include the use of immunosuppressants, capsid engineering, evading anti-capsid antibodies, modulating antigen presentation, and reducing cellular immune responses.

As mentioned above, the immunogenicity of AAV gene therapy may lead to safety concerns, which warrants further investigations and cautious study design. Yet, AAV can also be used for delivery of biologic therapeutics. In this case, the selection of capsids may be important, as previous clinical studies using AAV1 and AAV8 to express neutralizing antibodies for treating HIV achieved poor transgene expression. Davis-Gardner et al. performed a head-to-head comparison of five AAV capsids for delivering neutralizing antibodies in non-human primates, which generated key information for future therapeutic studies that aim to develop anti-HIV treatment using AAV. Key findings include: 1) successful antibody expression was achieved in spite of anti-drug antibodies; 2) AAV9 achieved higher antibody expression than AAV1, AAV8, AAV-NP22, and AAV-KP1; 3) P2A was preferred for separating heavy and light chains, in comparison to F2A and T2A.

In early 2023, the United States passed a new law eliminating the requirement that drugs under development must undergo animal testing before being given to humans. Apparently, the new trend is that the FDA commits to reducing the usage of laboratory animals in preclinical studies and relying more on alternative approaches. Therefore, a review article from Ramamurthy et al. is timely because it covers the immense potential of 3D organoids, microphysiological systems, and body-on-a-chip platforms to revolutionize preclinical testing. This article sheds light on critical questions regarding AAV gene therapy, which could be not answered by animal studies but can be at least partially addressed by studies in organoids and microphysiological systems.

## Author contributions

All authors have made a substantial, direct, and intellectual contribution to the work and approved it for publication.

